# A diffuse glioma with oligodendroglial‐like cells and extensive calcifications

**DOI:** 10.1111/bpa.13187

**Published:** 2023-07-06

**Authors:** Filippo Maria Martelli, Elena Marastoni, Valeria Barresi

**Affiliations:** ^1^ Department of Diagnostics and Public Health University of Verona Verona Italy

**Keywords:** *FGFR3::TACC3*, glioblastoma, *glioma*, PLNTY, *pTERT*

BOX 1 **Virtual glass slide**

Access at https://isn‐slidearchive.org/?col=ISN&fol=Archive&file=BPA‐23‐05‐CI‐132.svs

## 1 CLINICAL HISTORY AND IMAGING

**FIGURE 1:**
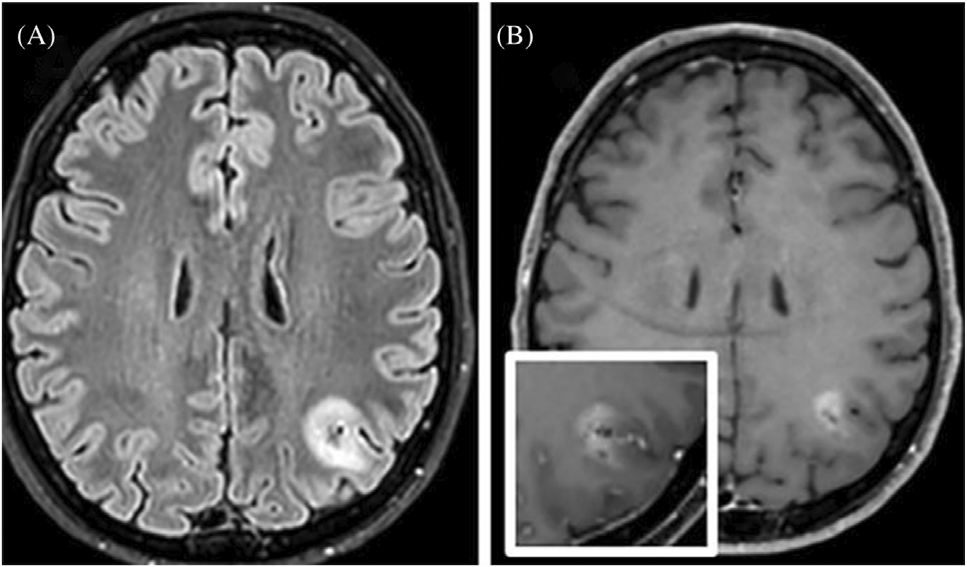
Magnetic resonance imaging showing hyperintensity on FLAIR (A) and heterogeneous hyperintensity on T1 (B) with focal nodular gadolinium contrast enhancement (inset).

Following a generalized tonic–clonic seizure during sleep, a 53‐year‐old woman underwent a computerized tomographic scan that revealed a calcified lesion of 15 mm in the left parietal lobe. On magnetic resonance imaging, the lesion was hyperintense on FLAIR, heterogeneously hyperintense on T1 and T2‐weighted images and showed focal nodular gadolinium contrast enhancement (Box 1, Figure 1). Treatment with antiepileptic drugs was initiated because of recurrent seizures. The lesion exhibited a modest increase in size over the following 10 months and was completely surgically removed 1 year after the initial radiological diagnosis. Three months after surgery, the patient was in good health with no radiological signs of recurrence, despite the lack of adjuvant treatment.

## 2 FINDINGS

**FIGURE 2:**
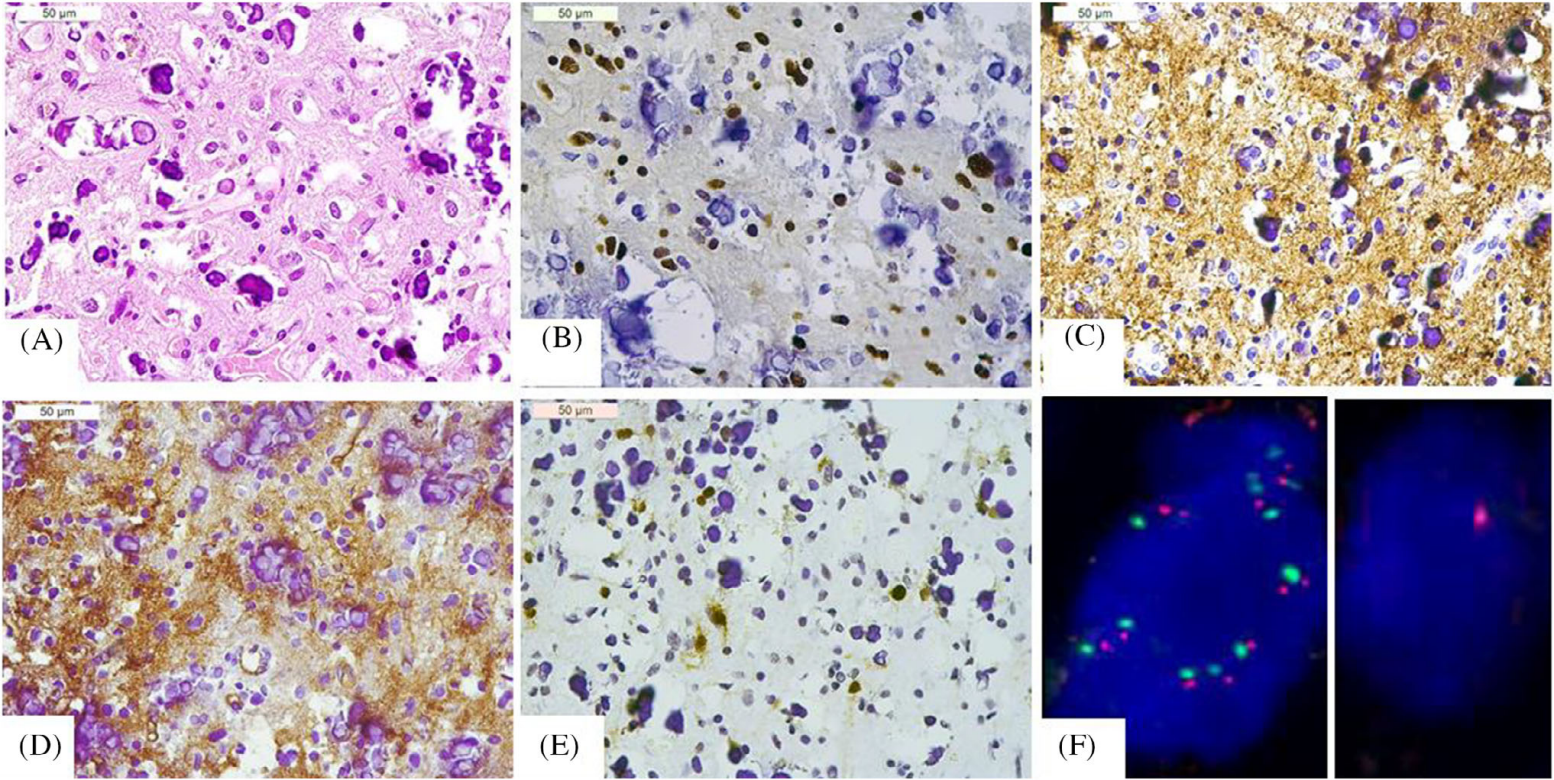
The tumor showed extensive microcalcifications and cells with oval, nuclei and a clear perinuclear halo (A), positive immunostaining for OLIG‐2 (B), GFAP (C), and CD34 (D), and intermingled Neu‐N‐positive neurons (E). FISH revealed multiple signals for the centromere of chromosome 7 (gains) (green probe) and the *EGFR* locus (red probe) (F, left), and a single signal for the centromere of chromosome 10 (loss) (F, right).

Histological examination revealed a tumor with extensive microcalcifications composed of neoplastic, oligodendroglial‐like cells with oval, hyperchromatic, mild pleomorphic nuclei, and a clear perinuclear halo (Figure 2). Mitoses, necrosis, or microvascular proliferation were absent.

By immunohistochemistry, the tumor was positive for OLIG‐2 and GFAP, showed CD34 extravascular stain (Figure 2), retained ATRX, and was negative for IDH1 p. R132H and BRAF p. V600E. Ki‐67 labeling index was 1%. NeuN immunostaining highlighted the intermingled nonneoplastic neurons (Figure 2). Next‐generation sequencing (NGS) confirmed the lack of mutations in the hotspots of *IDH1/2* and *BRAF* and revealed the mutation C228T in the *TERT* promoter (*pTERT*). RNA NGS showed the presence of *FGFR3* (exon 17)::*TACC3* (exon 4) fusion.

Fluorescent in situ hybridization revealed gains in chromosome 7 and loss in chromosome 10 (Figure 2) in the absence of *EGFR* amplification.

## 3 FINAL DIAGNOSIS

Diffuse glioma, histologically low grade, *IDH*‐wildtype, harboring *TERT* promoter mutation, chromosomes 7 gain/10 loss and *FGFR3::TACC3* fusion consistent with glioblastoma (GBM), *IDH* wild‐type, CNS WHO grade 4 (2021 WHO CNS tumor classification).

## 4 DISCUSSION

Diffuse gliomas with *FGFR3:TACC3* fusion display typical and recurrent morphological and immunohistochemical features, which consist of oligodendroglial‐like cells with monomorphous ovoid nuclei, nuclear palisading, thin parallel cytoplasmic processes, an endocrinoid network of thin capillaries, frequent microcalcifications, and CD34 immunostaining. The presence of some of these findings in our case prompted us to investigate *FGFR3::TACC3* fusion.

Diffuse gliomas with *FGFR3:TACC3* fusion do not represent a distinct nosological entity in the 2021 WHO classification of CNS tumors. Most reported cases had a high‐grade morphology, consisting of microvascular proliferation and necrosis [1, 2] and were considered to represent a subgroup of *IDH* wild‐type GBMs distinguished by prolonged overall survival. Within the minor proportion of diffuse gliomas with *FGFR3::TACC3* fusion that lack histological signs of malignancy, some cases are also devoid of the molecular features of GBMs, whereas others have chromosomes 7 gain/10 loss and/or *pTERT* mutation [1, 2], which supports a diagnosis of GBM, *IDH*‐wildtype, according to the 2021 WHO CNS classification, as in the present case. It has been suggested that these tumors may represent precursors of GBM or undersampled GBMs. Two studies have shown that the majority exhibit a distinct DNA methylation profile [1, 2]. Indeed, of 27 histologically diffuse low‐grade gliomas with *FGFR3::TACC3* fusion and *pTERT* mutation and/or chromosomes 7 gain/10 loss, only five cases had a calibration score above 0.90 for the methylation class “GBM IDH‐wildtype” using the 11b4 version of the Heidelberg classifier [1, 2]. Using the 12.5 version of the classifier, two tumors had calibration scores of 0.98 and 0.99 for the methylation class “ganglioglioma,” and none of the remaining cases had a calibrated score above 0.90 for any methylation classes [1, 2]. Therefore, the precise classification and grading of diffuse gliomas with *FGFR3::TACC3* fusion appear controversial, and a diagnosis more descriptive than GBM IDH‐wildtype may be adopted for these tumors. In the present case, the relatively indolent course over a 12‐month interval, the lack of frank progression and the stability 3 months after surgery despite the lack of adjuvant treatment, so far suggest a clinical behavior more consistent with a low‐grade diffuse glioma. Likewise, an overall survival exceeding 5 years has been reported in other patients with similar tumors. The morphological features observed in this case, along with the clinical history of seizures, might also suggest a diagnosis of polymorphous neuroepithelial tumor of the young (PLNTY), which is also characterized by oligodendroglioma‐like components, few (if any) mitotic figures, conspicuous calcifications, regional CD34 immunostaining, *IDH* wild‐type status, and fusions involving the *FGFR3* gene [3]. However, this CNS WHO grade 1 diffuse glioma generally occurs in children or young adults. In addition, all previously described cases with a diagnosis confirmed by DNA methylation profiling lacked the *FGFR3::TACC3* fusion and *pTERT* mutation.

## AUTHOR CONTRIBUTIONS

Filippo Maria Martelli and Elena Marastoni wrote the draft; Valeria Barresi reviewed, and edited the draft.

## CONFLICT OF INTEREST STATEMENT

The authors declare no conflicts of interest.

## ETHICS STATEMENT

All data related to this case are deidentified.

## Data Availability

The data that support the findings of this study are available from the corresponding author upon reasonable request.
